# Hepatoprotective Effect of Mixture of Dipropyl Polysulfides in Concanavalin A-Induced Hepatitis

**DOI:** 10.3390/nu13031022

**Published:** 2021-03-22

**Authors:** Dragana Arsenijevic, Bojana Stojanovic, Jelena Milovanovic, Aleksandar Arsenijevic, Milos Simic, Marija Pergal, Igor Kodranov, Olga Cvetkovic, Danilo Vojvodic, Elizabeta Ristanovic, Dragan Manojlovic, Marija Milovanovic, Nebojsa Arsenijevic

**Affiliations:** 1 Department of Pharmacy, Faculty of Medical Sciences, University of Kragujevac, 34000 Kragujevac, Serbia; menki@hotmail.rs; 2 Center for Molecular Medicine and Stem Cell Research, Faculty of Medical Sciences, University of Kragujevac, 34000 Kragujevac, Serbia; bojanastojanovic04@gmail.com (B.S.); jelenamilovanovic205@gmail.com (J.M.); aleksandar@medf.kg.ac.rs (A.A.); arne@medf.kg.ac.rs (N.A.); 3 Department of Pathophysiology, Faculty of Medical Sciences, University of Kragujevac, 34000 Kragujevac, Serbia; 4 Department of Histology, Faculty of Medical Sciences, University of Kragujevac, 34000 Kragujevac, Serbia; 5 Center for New Technologies, 11000 Belgrade, Serbia; milossim11@gmail.com; 6 Institute of Chemistry, Technology and Metallurgy—National Institute of the Republic of Serbia, University of Belgrade, 11000 Belgrade, Serbia; marijav@chem.bg.ac.rs (M.P.); olgacvet@chem.bg.ac.rs (O.C.); 7 Faculty of Chemistry, University of Belgrade, 11000 Belgrade, Serbia; ikodranov@chem.bg.ac.rs (I.K.); manojlo@chem.bg.ac.rs (D.M.); 8 Institute of Medical Research, Faculty of Medicine, Military Medical Academy, 11000 Belgrade, Serbia; vojvodic.danilo@gmail.com (D.V.); elizabet@eunet.rs (E.R.); 9 Department for Ecology and Chemical Technology, South Ural State University, 454080 Chelyabinsk, Russia

**Keywords:** dipropyl polysulfides, ConA hepatitis, anti-inflammatory activity, hepatoprotective effects

## Abstract

The main biologically active components of plants belonging to the genus *Allium,* responsible for their biological activities, including anti-inflammatory, antioxidant and immunomodulatory, are organosulfur compounds. The aim of this study was to synthetize the mixture of dipropyl polysulfides (DPPS) and to test their biological activity in acute hepatitis. C57BL/6 mice were administered orally with DPPS 6 h before intravenous injection of Concanavalin A (ConA). Liver inflammation, necrosis and hepatocytes apoptosis were determined by histological analyses. Cytokines in liver tissue were determined by ELISA, expression of adhesive molecules and enzymes by RT PCR, while liver mononuclear cells were analyzed by flow cytometry. DPPS pretreatment significantly attenuated liver inflammation and injury, as evidenced by biochemical and histopathological observations. In DPPS-pretreated mice, messenger RNA levels of adhesion molecules and NADPH oxidase complex were significantly reduced, while the expression of SOD enzymes was enhanced. DPPS pretreatment decreased protein level of inflammatory cytokines and increased percentage of T regulatory cells in the livers of ConA mice. DPPS showed hepatoprotective effects in ConA-induced hepatitis, characterized by attenuation of inflammation and affection of Th17/Treg balance in favor of T regulatory cells and implicating potential therapeutic usage of DPPS mixture in inflammatory liver diseases.

## 1. Introduction

Liver inflammatory diseases, caused by viral infections, alcohol abuse, drugs, or autoimmune reactions, are still one of the major threats to human health. ConA-induced hepatitis in mice is primarily mediated by T cells and has been widely used as a model for acute or fulminant immune-mediated liver diseases [1,2]. After intravenous injection, ConA binds to liver sinusoidal endothelial cells (SEC) and induces their damage [3]. Damaged SEC allow ConA to bind Kupffer cells, which induce polyclonal activation of T cells. T cells, but also other immune cells such as natural killer (NK) cells, natural killer T (NKT) cells, are recruited into the liver, secrete inflammatory cytokines, tumor necrosis factor alpha (TNF-α), interleukin-17, interferon gamma (IFN-γ) which consequently induce massive apoptotic or necrotic death of hepatocytes and severe liver inflammation accompanied by highly elevated levels of transaminases in serum [1,4,5,6,7], and in turn stimulate Kupffer cells and inflammatory monocytes to produce more inflammatory cytokines, TNF-α and IL-1β [8]. Production of inflammatory cytokines and hepatocytes death in ConA-induced hepatitis is enhanced by superoxide anions released by stimulated Kupffer cells and hepatocytes [9].

Plants of the genus *Allium*, to which garlic belongs, have been used in diet and folk medicine and phytotherapy for centuries. Hepatoprotective effects of garlic extract have been shown in mice exposed to ethanol [10]. The main biologically active components of garlic are organosulfur compounds (OSCs), not present in the active form in untreated garlic. These active compounds are released during garlic tissue damage when S-alk(en)yl cysteine sulfoxides are converted to their respective thiosulfinates or propanethial-S-oxide by the action of the enzyme alliinase [11]. Thiosulfinates can be further decomposed to form additional sulfur constituents including diallyl, methyl allyl, and diethyl mono-, di-, tri-, tetra-, penta-, and hexasulfides [11]. OSCs attenuate the release of proinflammatory cytokines IL-1β, TNF-α, IL-6 from LPS-stimulated RAW 264.7 macrophages by inactivating the transcription factor NF-κB, and inhibit the production of nitric oxide and prostaglandin E2 by regulating the expression of inducible nitric oxide synthase, iNOS and COX-2, respectively [12,13,14,15]. Aged garlic extracts that contain active OSCs enhance expression of antioxidant enzymes such as superoxide dismutase (SOD) in endothelial cells and thus protect them from oxidant injury by ROS [16]. Anti-inflammatory and hepatoprotective effects of OSCs have been shown in nonalcoholic steatohepatitis [17,18].

It is known that a higher number of sulfur atoms in organosulfur compounds (DATS > DADS > DAS) is associated with significantly higher antimicrobial, antioxidant and anticancer activity [18,19,20], but previous studies have been mostly focused on the research of garlic extracts and diallyl mono-, di-, tri and tetra-sulfides [21]. Biological effects of saturated derivates of OSCs, dipropyl polysulfides, are also poorly explored.

Since the OSCs compounds with more than six sulfur atoms in their structure are undetectable in garlic and onion and are expected to exhibit a significantly stronger biological activity [22], we aimed to synthetize the mixture of dipropyl polysulfides with a higher number of sulfur atoms in their structure and to explore their biological effect in vivo in mouse model of ConA-induced acute liver damage. We have shown that our mixture contains dipropyl polysulfides (DPPS) with eleven sulfur atoms. We demonstrated that pretreatment with DPPS significantly alleviated ConA-induced hepatitis in mice. DPPS pretreatment significantly attenuated liver inflammation and hepatocytes death, serum levels of aminotransferases, concentration of inflammatory cytokines in the liver tissue and enhanced expression of hepatoprotective enzymes, SODs, and accumulation of T regulatory cells in the livers of ConA-injected mice. Thus, our data implicate that DPPS mixture may be considered as a potential therapeutic for acute immune-mediated liver injury.

## 2. Materials and Methods

### 2.1. Chemicals

Acetonitrile (>99.9%, Sigma-Aldrich HPLC grade), tetrahydrofuran (>99.9%, Sigma Aldrich HPLC grade) and water (HPLC Plus grade water, Sigma-Aldrich, Saint Louis, MO, USA) were used for HPLC analysis. Syringe filters (25 mm, PTFE membrane 0.45 μm) were obtained from Agilent Technologies.

### 2.2. Synthesis of the Mixture of Dipropyl Polysulfides (DPPS)

Potassium polysulfide (p.a., Carl Roth) with slight excess of sulfur was placed in a flask where was previously placed five times volume of tetrahydrofuran (≥99.5%, for synthesis Carl Roth), in which was added 2% *v*/*v* of distilled water. Reflux condenser was placed on the flask and reaction mixture was refluxed for 12 h. After that solution was left to cool down and in cold solution, an equivalent amount of propyl bromide (99% Acros Organics) was added dropwise with vigorous stirring. After the addition reaction mixture was again refluxed for 12 h. Obtained mixture was placed in a separation funnel and organic layer was separated and rinsed three times with 10% solution of sodium chloride. Organic solvent was evaporated and, in the flask, remains mixture of polysulfides. Length of sulfur chain depends on reflux time in the first step of the synthesis.

### 2.3. Analytical Procedure

High-performance liquid chromatography (HPLC) with photodiode array detection (DAD) was performed using Thermo Ultimate 3000 RS on a Supelcosil^TM^ LC-18-DB analytical column (150 mm × 4.6 mm, 3 µm; Sigma-Aldrich) at 37 °C. The mobile phase consisted of water as component A and 3% of tetrahydrofuran/97% of acetonitrile as component B. The chromatographic elution was conducted at a flow rate of 1.0 mL/min in gradient mode: 0.0–10.0 min from 30% to 70% B, 10.0–35.0 min 70% B, 35.0–40.0 min from 70% to 30% B, 40.0–50.0 min 30% B. The detector was set at 240 nm. Injection volume was 20 µL. Data analysis was performed with software Chromeleon, v6.8 (ThermoFisher Scientific, Bremen, Germany).

The same eluent gradient, used for HPLC analysis, was used for preparative HPLC in order to separate compounds that are contained in the synthesized mixture. Separation of compounds was monitored using DAD as a detector and eluent with separated compounds was collected in separate glass tubes. Combined solutions for each of the separated compounds was evaporated to dryness on a water bath in ceramic dishes. After evaporation, obtained substances, with a higher number of sulfur atoms than 7, were analyzed using elemengtal analyzer Elementar Vario-EL III CHNS-O (Hannau, Germany). The results of these analyses were: compound 8 (C 21.12%, H 4.06%, N 0.00%, S 74.82%), compound 9 (C 19.18%, H 3.78%, N 0.00%, S 77.04%), compound 10 (C 17.80%, H 3.41%, N 0.00%, S 78.79%), compound 11 (C 16.49%, H 3.11%, N 0.00%, S 80.40%). Based on these results, which correspond to dipropyl polysulfides with 8, 9, 10 and 11 sulfur atoms, it can be concluded that we managed to obtain dipropyl polysulfides (DPPS) with eleven sulfur atoms. Polysulfides with a lower number of sulfur atoms were identified by comparison with garlic oil (Sigma Aldrich) using HPLC-DAD.

### 2.4. Mice, Hepatitis Induction and DPPS Treatment

Male C57BL/6 mice weighing approximately 18 ± 22 g (6–8 weeks of age) were used in the experiments. The mice were housed in the Center for Molecular Medicine and Stem Cell Research, Faculty of Medical Sciences, University of Kragujevac. All animal procedures were approved by the Ethics Committee of Faculty of Medical Sciences, University of Kragujevac and conducted in accordance with the National Institutes of Health guidelines for humane treatment of laboratory animals. The 269 animals were randomly divided into untreated, ConA (12.5 mg/kg in 200 µL of saline intravenously), DPPS (20 µL of 50% mixture solution, orally), DPPS + ConA (20 µL of 50% mixture solution, orally, 6 h before intravenous administration of 12.5 mg/kg ConA) groups. One single oral administration corresponded to 12.1 mg of DPPS and the dose was chosen according to previous reports about protective roles of orally administered OSCs in inflammatory diseases and cancer [23]. The number of the groups varied according to the experiment. For analysis of orally administered DPPS on phenotype of immune cells in the livers, mice were divided into two groups: 1. untreated and 2. DPPS-treated with 8 mice per group. For analysis of serum transaminases and histology, mice were divided into four groups: 1. untreated with 7 mice per group, while groups 2. DPPS, 3. DPPS + ConA, and 4. ConA had 28 mice per group and every of these three groups was further subdivided into four subgroups for four different times of sacrificing. For TUNEL analysis, mice were divided into four groups: 1. untreated, 2. DPPS, 3. DPPS + ConA, and 4. ConA with 6 mice per group. For PCR analysis, mice were divided into three groups: 1. DPPS, 2. DPPS + ConA-injected, and 3. ConA with 5 mice per group. For ELISA assay, mice were divided into four groups: 1. untreated, 2. DPPS, 3. DPPS + ConA, and 4. ConA with 14 mice per group, but every group was further divided into two groups for two different times of sacrificing. For flow cytometric analysis of liver mononuclear cells, mice were divided into four groups: 1. untreated, 2. DPPS-treated, 3. DPPS-treated and ConA-injected, and 4. ConA-injected with 6 mice per group. The mice were sacrificed 2, 4, 6, 8, 12, and 24 h after ConA injection. Blood samples were obtained, and the liver was collected simultaneously.

### 2.5. Serum Levels of Transaminases

Serum levels of aspartate aminotransferase (AST) were measured 2, 4, 12, and 24 h after ConA injection by spectrophotometric method using the automated biochemistry analyzer Olympus AU 400 (Olympus Diagnostica GMBH, Hamburg, Germany) and Olympus AU reagents, according to the manufacturer’s instructions, expressed in U/L.

### 2.6. Liver Histology and TUNEL Staining

Livers were fixed with 4% paraformaldehyde and embedded in paraffin. Next, they were cut on a microtome to obtain 5 μm thick sections and the sections were stained with hematoxylin and eosin (H&E) and examined under low-power light microscopy (BX51; Olympus) equipped with digital camera. Death of hepatocytes was determined by TUNEL (terminal deoxynucleotidyl transferase-mediated dUTP nickend labeling) staining of liver sections. Paraffin-embedded liver tissue sections were stained with in situ Cell Death Detection Kit, POD (Roche) according to the manufacturer’s instructions. DAB (3,3′-diaminobenzidine) was added as a substrate for peroxidase in order to obtain typical brown coloration of the nuclei. Slides were counterstained with hematoxylin and photomicrographed with a digital camera mounted on light microscope. The TUNEL-positive nuclei (brown) were quantified under magnification 400× in five randomly fields and the data were summarized as the mean number of positive cells.

### 2.7. Cytokine Measurements

After extirpation, a portion of the liver weighing 100 mg was homogenized in 0.5 mL PBS. Obtained homogenates were centrifuged at 14,000× *g* for 10 min at 4 °C and supernatants were transferred to clean microcentrifuge tubes and stored at −20 °C. Cytokine levels in liver supernatants were determined using mouse Duoset enzyme-linked immunosorbent assay (ELISA) kits for TNF-α, IL-1β, IL-6, IL-10, IL-12, and IL-17 (R&D Systems) according to the manufacturer’s instructions.

### 2.8. Isolation of Hepatic Mononuclear Cells and Flow Cytometry

The isolation of liver-infiltrating inflammatory mononuclear cells was conducted as previously described [24]. Mononuclear cells isolated from liver were resuspended in FACS buffer (PBS with 5 mM EDTA and 0.2% BSA) incubated with the fluorochrome-conjugated anti-mouse CD4, CD8, CD69, CD25, FoxP3, F4/80, CD86, CD206, CD11c antibodies or their respective isotype controls. For intracellular staining, cells were incubated for 4 h at 37 °C in the presence of 50 ng/mL phorbol 12-myristate 13-acetate (PMA) (Sigma-Aldrich), 1 μg/mL ionomycin (Sigma-Aldrich) and Golgi Stop (BD Biosciences, San Jose, CA, USA). After incubation with PMA and ionomycin, cells were fixed and permeabilized by BD Cytofix/Cytoperm buffers (BD Biosciences, San Jose, CA, USA). After fixation and permeabilization, cells were incubated with anti-mouse IFN-γ, IL-17 and IL-10 antibodies. Isotype controls were included to set gates. Expression of cell surface and intracellular antigens was analyzed with FACSCalibur Flow Cytometer (BD Biosciences). Flow cytometric analysis was conducted with FlowJo Software (Tree Star, Phoenix, AZ, USA).

### 2.9. RNA Extraction and Real-Time qRT-PCR

Total RNA from mouse liver tissue was extracted using TRIzol (Invitrogen, Carlsbad, CA, USA). Total RNA (2 μg) was reverse-transcribed to cDNA using RevertAid H Minus First Strand cDNA Synthesis Kit (Thermo Fisher Scientific, Vilnius, Lithuania). qRT-PCR was performed using Luminaris Color HiGreen qPCR Master Mix (Thermo Fisher Scientific) and miRNA specific primers presented in Table 1 in a Mastercycler ep realplex (Eppendorf, Hamburg, Germany). Relative expression of genes was calculated according to the formula 2^−(Ct−Ctactin)^, where C_t_ is the cycle threshold of the gene of interest and C_tactin_ is the cycle threshold value of the housekeeping gene (GAPDH).

### 2.10. Statistical Analysis

The data are presented as mean ± SD or mean ± SEM. Statistical significance was determined by Independent sample Student *t*-test and ANOVA, and, where appropriate, Mann-Whitney *U*-test or Kruskal-Wallis. Statistical significance was assumed at *p* < 0.05. Statistical analyses were performed using SPSS 13.0.

## 3. Results

### 3.1. Mixture of Dipropyl Polysulfides Stimulates Activation and Regulatory Phenotype of Antigen Presenting Cells in the Liver

**Figure 1:**
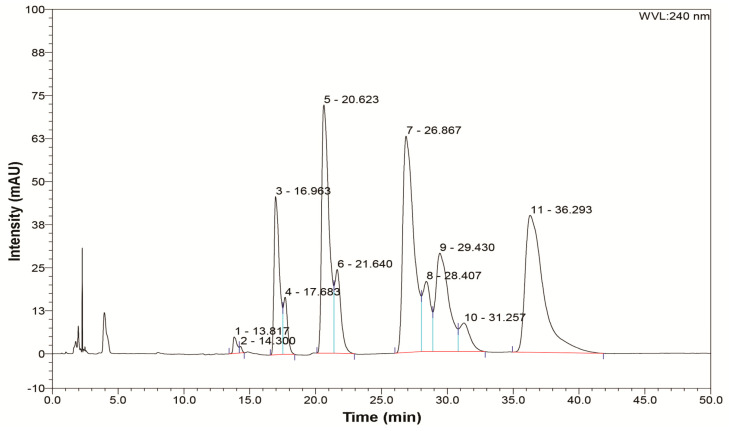
HPLC chromatogram of different dipropyl polysulfides in synthesized mixture.

Our mixture contains polysulfides that are present in garlic and onion like dipropyl disulfide, dipropyl trisulfide, dipropyl tetrasulfide, but also polysulfides with a higher number of sulfur atoms like dipropyl undecasulfide. Content in our mixture is different for every polysulfide, e.g., higher percent of dipropyl pentasulfide (20.99%), dipropyl heptasulfide (24.27%) and dipropyl undecasulfide (25.39%) was obtained, while medium content was obtained for dipropyl trisulfide (8.48%), dipropyl tetrasulfide (4.26%), dipropyl hexasulfide (5.11%), dipropyl octasulfide (4.62%), dipropyl nonasulfide (5.11%) and minimal content for dipropyl disulfide (0.51%) and dipropyl decasulfide (1.26%) (Figure 1).

**Figure 2:**
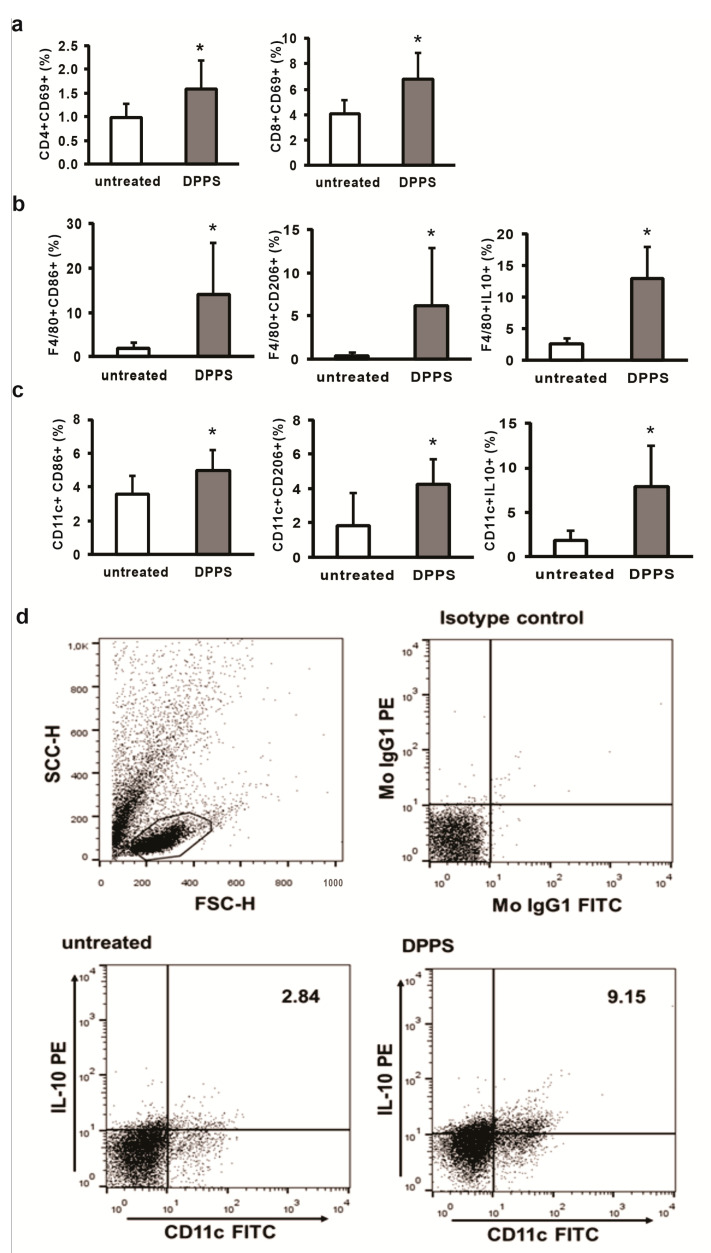
Pretreatment with dipropyl polysulfides (DPPS) increases percentage of activated and regulatory macrophages and DCs in the liver. (**a**) Percentage of activated, CD69-positive, CD8+ and CD4+ cells, (**b**) F4/80+ macrophages and (**c**) CD11+ dendritic cells, expressing marker of classical (CD86) and alternative activation (CD206) and anti-inflammatory cytokine IL-10, determined by flow cytometry of mononuclear cells isolated from the livers of untreated mice and 6 h after oral treatment with DPPS, and gating strategy for detection of double-positive cells (**d**). Data are presented as mean + SD (* *p* < 0.05; two-tailed, unpaired Student’s *t*-test).

After oral administration, dipropyl disulfides (DPDS) were rapidly metabolized in the liver and it appears that orally administered DPDS have no systemic bioavailability [25]. Liver is also the place of storage of sulfur containing metabolites of DPDS [25]. All DPDS metabolites are also identified in rat liver perfused with DPDS [26]. Taking into account these facts and known various immunodulatory activities of fresh garlic derivatives and aged garlic extract, a product of the prolonged extraction of fresh garlic soaked in aqueous/ethanol solution, rich in organosulfur compounds [27], we firstly analyzed the effect of orally administered compound on phenotype of immune cells in the livers. Mixture of dipropyl polysulfides do not change cellular composition of immune cells in the liver, six hours after oral administration; however, changes of phenotype have been noticed (Figure 2). In the livers of orally administered polysulfides, significantly higher percentages of activated, CD69-positive CD4+ and CD8+ cells were noticed (Figure 2a). A higher percentage of F4/80+ macrophages expressing markers of classical (CD86), but also alternative (CD206) activation, has been detected in the livers of DPPS-treated mice (Figure 2b). Further, DPPS treatment significantly enhanced percentage of regulatory IL-10-expressing macrophages (Figure 2b). The same effect of DPPS treatment on dendritic cells phenotype was also observed. Significantly higher percentages of activated (CD86- and CD206-positive) and IL-10-expressing CD11c+ dendritic cells were noticed in the livers of DPPS-treated mice (Figure 2c).

### 3.2. Mixture of Dipropyl Polysulfides Exerts Hepatoprotective Effects

**Figure 3:**
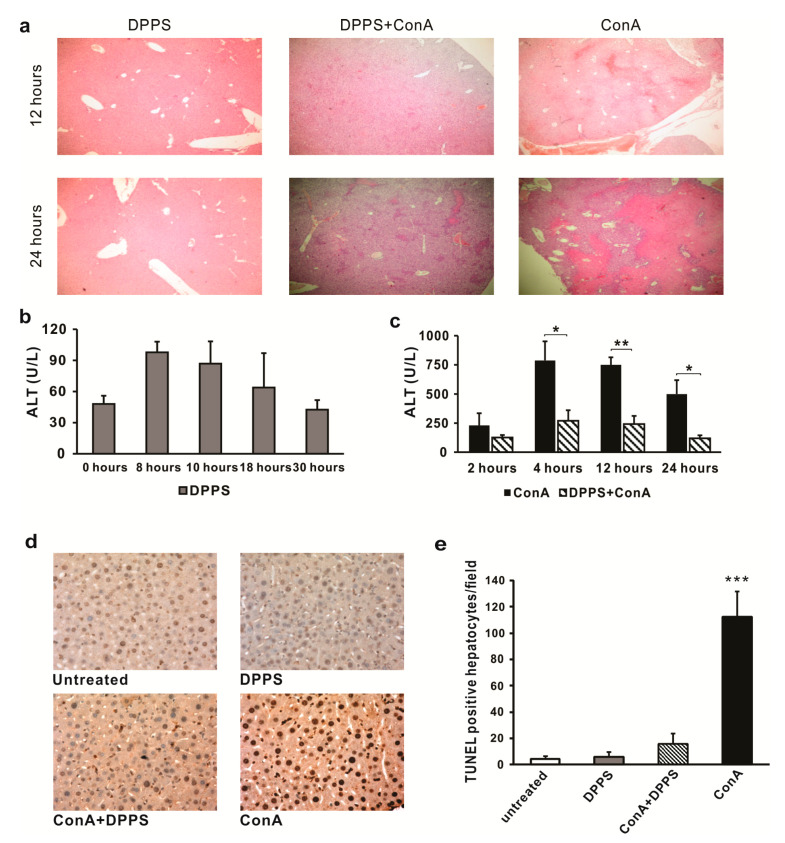
Pretreatment with DPPS attenuates markers of liver damage in ConA-induced acute hepatitis. DPPS pretreatment shows hepatoprotective effects in ConA-induced hepatitis. C57BL/6 mice were intravenously injected with 12.5 mg/kg of Concanavalin A. DPPS was administered orally (20 µL of 50% mixture solution), six hours before ConA injection. Livers were analyzed 12 and 24 h after ConA injection. (**a**) Representative H&E staining of paraffin-embedded liver sections, magnification 100×. (**b**) ALT levels determined in the serum 0, 8, 10, 18, and 30 h after oral administration of DPPS. (**c**) Serum levels of ALT determined 2, 4, 12, and 24 h after ConA injection (*n* = 7). (**d**) TUNEL staining of liver sections 6 h after ConA injection, magnification 400×. (**e**) Quantitative analysis of cell death rate: TUNEL-positive nuclei (brown) were counted in five random fields, and the data were summarized as the mean number of positive cells+SD (*** *p* < 0.001; ** *p* < 0.005; * *p* < 0.05; two-tailed, unpaired Student’s *t*-test).

Since it has been noticed that orally administered dipropyl polysulfides induce immunosuppressive environment in the liver, potential hepatoprotective effects of this mixture were tested in ConA-induced acute hepatitis. Significantly reduced liver damage has been observed in mice treated with DPPS six hours before intravenous administration of ConA (Figure 3). In the livers of DPPS-pretreated mice, only inflammatory focci were noticed 12 h after ConA administration, while significant areas of necrosis were found in the livers of ConA mice (Figure 3a). This difference between DPPS-treated and -untreated mice was even higher 24 h after ConA application (Figure 3a). There were inflammatory and few small necrotic focci in the livers of DPPS-treated mice, 24 h after ConA injection, while confluent necrotic areas involving the majority of the liver tissue were seen in untreated mice (Figure 3a). No tissue damage was noticed in the livers of DPPS-only-treated mice (Figure 3a). Treatment with DPPS only did not increase serum level of ALT (Figure 3b), but significant attenuation of liver damage by DPPS was confirmed by analysis of ALT levels in the serum of ConA-treated mice. Slight increase of ALT level in the sera of DPPS-treated mice was noticed 8 and 10 h after DPPS treatment, but it returned to baseline levels after 18 h (Figure 3b). ALT serum levels were significantly lower in DPPS-pretreated, in comparison with untreated mice, 4, 12, and 24 h after ConA injection (Figure 3c).

In line with previous report that ConA treatment induces massive hepatocyte apoptosis [28], massive apoptosis has been detected by TUNEL assay in the livers of ConA-treated mice. As it is shown in Figure 3d,e, DPPS pretreatment significantly prevented apoptosis of hepatocytes. There is no increase of the number of apoptotic hepatocytes in the livers of mice treated only with DPPS (Figure 3d). DPPS pretreatment significantly (*p* < 0.001) decreases the number of TUNEL-positive hepatocytes in ConA-treated mice (Figure 3e).

**Figure 4:**
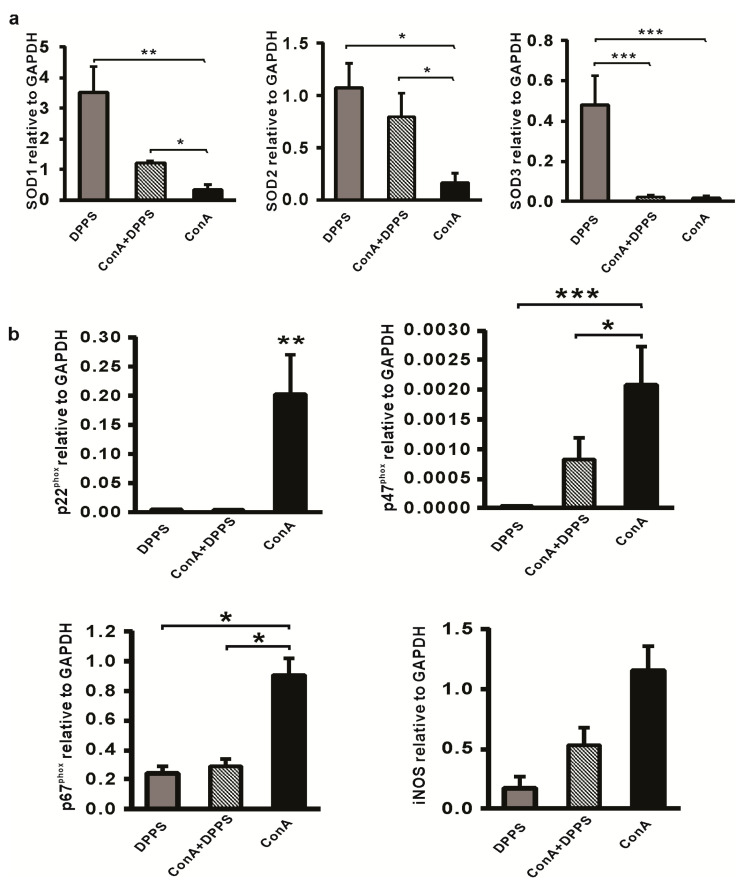
Pretreatment with DPPS shows hepatoprotective effects in ConA-induced hepatitis. mRNA levels of (**a**) SOD1, SOD2, SOD3 and (**b**) p22^phox^, p47^phox^, p67^phox^, iNOS in livers determined using real-time qRT-PCR with GPDH as an internal control, 8 h after infection (*n* = 5). Data are presented as mean + SE (*** *p* < 0.001; ** *p* < 0.005; * *p* < 0.05).

Since decreased expression of inducible NO synthase (iNOS) [29] and NADPH oxidase (nicotinamide adenine dinucleotide phosphate oxidase) complex [30] mediated by organosulfur compound is associated with anti-inflammatory and tissue protective effects, while attenuation of inflammatory processes in the liver is associated with increased presence of superoxide dismutase [31], we next analyzed expression of these enzymes in the livers of mice treated with DPPS. Expression of intracellular anti-oxidative enzymes SOD1 (*p* < 0.01) and SOD2 (*p* < 0.05) and extracellular SOD3 (*p* < 0.001) in the liver tissue of DPPS-treated mice is significantly higher in comparison with expression of these enzymes in the liver tissue eight hours after ConA injection (Figure 4a). Significantly higher expression of SOD1 and SOD2 mRNAs (*p* < 0.05) were also found in the livers of DPPS-pretreated ConA mice in comparison with the group of ConA mice, while increased expression of SOD3 was not noticed in the group of DPPS-pretreated ConA mice (Figure 4a). As it is shown in Figure 4b, DPPS pretreatment significantly attenuates expression of enzymes of NADPH oxidase complex, p22^phox^, p47^phox^, and p67^phox^ in the liver tissue obtained 8 h after ConA injection. Expression of iNOS is also decreased in the livers of DPPS-pretreated mice compared with ConA-only-injected mice, but this difference did not reach statistical significance (Figure 4b).

### 3.3. Anti-Inflammatory Activity of Mixture of Polysulfides in ConA-Induced Hepatitis

**Figure 5:**
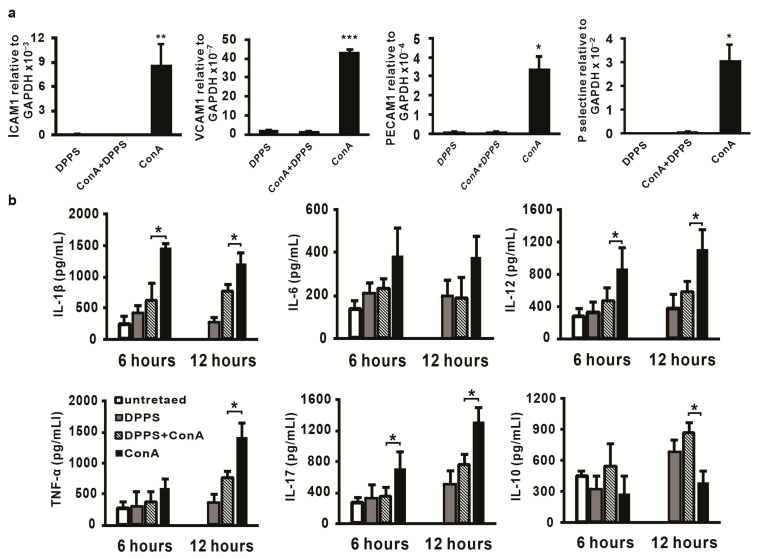
DPPS attenuates liver inflammation induced by ConA. (**a**) ICAM-1, VCAM-1, PECAM-1, and P-selectine mRNA expression in livers determined using real-time qRT-PCR with GPADH mRNA as an internal control, 12 h after infection (*n* = 5) presented as mean + SE. (**b**) Concentration of IL-1β, IL-6, IL-12, IL-17, TNF-α, and IL-10 in the liver tissue homogenate 6 and 12 h after ConA injection determined by ELISA presented as mean + SD (*** *p* < 0.001; ** *p* < 0.005; * *p* < 0.05).

It is known that enhanced expression of adhesion molecules plays a critical role in inflammatory processes and also in ConA-induced hepatitis [32]. Thus, the next analysis was done in order to analyse changes in ICAM-1, VCAM-1, PECAM-1, and P-selectin mRNA expression in the livers of mice treated with DPPS and ConA. As shown in Figure 5a, the mRNA levels of all tested genes were significantly lower in the livers of DPPS-pretreated mice in comparison with mRNA levels in the liver tissue of mice injected only with ConA. Furthermore, mRNA levels of ICAM-1, VCAM-1, PECAM-1, and P-selectin in the livers of DPPS-pretreated ConA mice were not higher in comparison with the liver tissue of DPPS-only-treated mice (Figure 5a).

Since ConA injection is followed with significant production of inflammatory cytokines in the liver [33], the influence of DPPS treatment on concentration of inflammatory cytokines and anti-inflammatory IL-10 in liver tissue homogenates was next tested. DPPS treatment did not alter production of IL-6 in the liver (Figure 5b). Production of IL-1β, IL-12, and IL-17 was significantly attenuated in the livers of DPPS-pretreated mice, 6 and 12 h after ConA injection (Figure 5b). Significant production of TNF-α in the liver was noticed 12 h after ConA injection, and at this point in time, DPPS pretreatment significantly reduced production of this cytokine (Figure 5b). On the other hand, production of anti-inflammatory IL-10 was significantly enhanced in the livers of DPPS-pretreated ConA-injected mice (Figure 5b). Further, concentration of IL-10 was significantly higher in the livers of mice treated only with DPPS in comparison with untreated (Figure 5b).

### 3.4. Mixture of Polysulfides Enhances Percentage of Regulatory T Cells and Attenuates Percentage of IL-17-Producing Cells in the Liver

**Figure 6:**
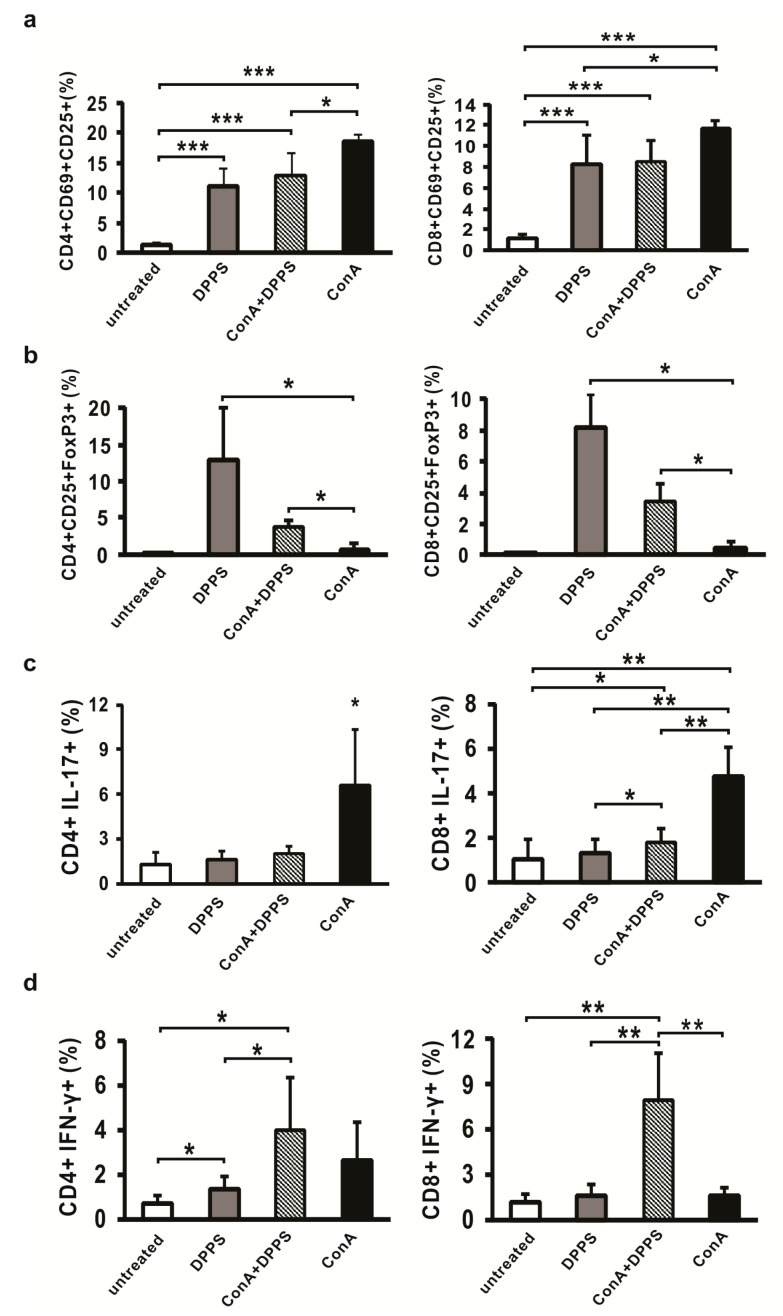
Effects of DPPS on phenotype of lymphocytes in the livers of ConA-treated mice. Percentages of activated CD25+CD69+ (**a**), regulatory CD25+FoxP3+ (**b**), IL-17+ (**c**) and IFN-γ+ (**d**) CD4+ and CD8+ cells determined by flow cytometry of mononuclear cells isolated from the livers 12 h after ConA injection (*n* = 6). Data are presented as mean + SD (*** *p* < 0.001; ** *p* < 0.005; * *p* < 0.05).

ConA administered intravenously induces recruitment of inflammatory lymphocytes in the liver which, through their inflammatory cytokines, mediate hepatocyte death [1]. In order to explore the effect of DPPS pretreatment on profiles of inflammatory cells in the livers after ConA administration, mononuclear cells isolated from the livers were analyzed by flow cytometry. Significantly enhanced percentage of activated lymphocytes in the livers of mice treated with DPPS is maintained even 14 h after DPPS treatment (Figure 6a). Further, percentage of activated CD25+CD69+CD8+ cells is very similar in the groups of DPPS-treated, DPPS-pretreated ConA-injected, and ConA-injected mice, while percentage of activated CD25+CD69+CD4+ cells was significantly higher in the group of ConA-injected mice than in other groups (Figure 6a). However, on the other hand, percentages of regulatory CD4+CD25+FoxP3+ and CD8+CD25+FoxP3+ cells are significantly higher in the livers of DPPS-pretreated mice in comparison with the percentage of these cells in the group of ConA mice (Figure 6b). Percentage of regulatory CD4+ and CD8+ cells is also significantly higher in the livers of mice treated only with DPPS in comparison with untreated mice and mice treated only with ConA (Figure 6b). No significant difference was observed between DPPS-only-treated and DPPS-pretreated ConA-injected mice (Figure 6b). DPPS pretreatment significantly decreases the percentage of IL-17-expressing CD4+ and CD8+ cells (Figure 6c), while enhances the percentage of IFN-γ-positive CD4+ and CD8+ cells (Figure 6d) in the livers, 12 h after ConA injection.

## 4. Discussion

In the present study, we investigated the effect of the mixture of newly synthetized dipropyl polysulfides, known as biologically active ingredients of garlic [21], on ConA-induced hepatitis. The results showed that DPPS pretreatment, 6 h prior to ConA injection, markedly downregulated serum ALT levels and inhibited hepatocyte apoptosis/necrosis. Further, DPPS pretreatment significantly reduced the mRNA levels of adhesion molecules like ICAM-1, VCAM-1, PECAM-1, and P-selectin and protein levels of inflammatory cytokines, while enhanced expression of anti-inflammatory IL-10 in the liver. Our results also indicate that mixture of dipropyl polysulfides in the liver enhances percentage of activated lymphocyte with regulatory phenotype, while decreases percentage of inflammatory IL-17- and IFN-γ-expressing CD4+ and CD8+ cells. Tested mixture also decreased expression of NADPH oxidase complex enzymes and enhanced expression of SOD enzymes in the liver. Therefore, our findings suggest that mixture of dipropyl polysulfides has a protective effect on ConA-induced hepatitis.

Intravenous administration of well-known T cell mitogen, ConA, induces massive influx of lymphocytes in the liver followed by abundant liver necrosis [34]. ConA-induced hepatitis is a well-known mouse model of acute or fulminant immune-mediated liver damage [1,2]. Adhesion molecules play important roles in inflammatory and immune responses in ConA-induced hepatitis. Studies with ICAM-1 knockout mice revealed that ICAM-1 plays a role in the development of ConA-induced hepatitis by regulating leukocyte infiltration and subsequent cytokine production [32]. A similar finding was reported for P-selectin, P-selectin-deficient mice developed significantly milder ConA-induced hepatitis [35]. Expression of VCAM-1 is significantly enhanced in mice that develop hepatitis after ConA injection [36], while the role of PECAM-1 in leukocyte transmigration during the liver inflammation is well known [37]. In this study, we have found that mixture of dipropyl polysulfides significantly reduced mRNA expression of ICAM-1, VCAM-1, PECAM-1, and P-selectin (Figure 5a). Our result is in line with previous reports on decreased expression of adhesion molecules in the liver of mice treated with substances that alleviate ConA-induced liver damage [38,39,40]. Increased expression of adhesive molecules in liver tissue is necessary for the process of inflammation, influx of the leukocytes in the liver, followed by cytokine production [32]. In accordance with decreased expression of adhesion molecules in the livers of DPPS-treated ConA-injected mice, we also found decreased expression of inflammatory cytokines, IL-1β, TNF-α, IL-12, IL-17, in the liver tissues of these mice (Figure 5b). ConA-induced hepatitis is associated with the release of large amounts of proinflammatory cytokines that lead to hepatocytes injury, apoptosis and necrosis [41,42,43,44]. Among these cytokines, TNF-α and IL-1β could be the critical mediators for development of ConA-induced hepatitis, since TNF-α-deficient mice [4] and NLRP3- and caspase-1-deficient mice that do not produce IL-1β [41] are almost protected from ConA-mediated liver damage. Previous study indicates that garlic sulfur compound, allicin, attenuates TNF-α production and adhesion molecules expression in ConA-induced hepatitis and thus attenuates liver damage [45]. Consistent with previous experimental data, here we have shown that DPPS pretreatment significantly reduced TNF-α and IL-1β levels in the livers of ConA-treated mice (Figure 5b), expression of adhesive molecules (Figure 5a) and thus probably contributed to attenuation of liver damage (Figure 3) and to protective effects of DPPS on ConA-induced hepatitis.

Kupffer cells and hepatocytes in hepatitis induced by ConA produce superoxide, a molecule that promotes production of inflammatory cytokines and hepatocyte apoptosis [9]. The main source of reactive oxygen species are reactions mediated by NADPH oxidases, a complex enzyme consisting of at least six subunits, the membrane bound gp91^phox^ and p22^phox^homodimer, the cytoplasmic complex of p40^phox^, p47^phox^ and p67^phox^ [46]. SOD is an enzyme that, by scavenging superoxide anions, plays the main role in maintaining the balance between oxidation and antioxidation and protects the cells from injury [47]. Here, we have found that DPPS pretreatment significantly increased SOD1, SOD2, and SOD3 expression (Figure 4a) and significantly reduced expression of p22^phox^, p47^phox^ and p67^phox^ (Figure 4b) in ConA-injected mice. These results are in line with previous reporting that tissue protective effects of S-allylcysteine are mediated by enhancing expression of SOD, but also by reducing the expression of NADPH oxidase p22^phox^ and gp91^phox^ [48]. Also, hepatoprotective effects of sulfur compound, allicin, in fish model of liver injury seems to be mediated by enhancing the activity of SOD [49]. These findings, together with previously discussed results that DPPS downregulates expression of proinflammatory cytokines (Figure 5b), support the antiapoptotic effect of DPPS in vivo (Figure 3d,e).

Regulatory T cells suppress inflammation, regulate immune system activity, and can contribute to attenuation of immune-mediated diseases [50]. It has been shown that adoptive transfer of Tregs significantly reduces liver injury in ConA-induced hepatitis by modulating the balance between Treg and Th17 cells [51]. We have found that DPPS pretreatment significantly enhances percentages of FoxP3+ regulatory CD4+ and CD8+ cells in the livers of mice with ConA-induced hepatitis (Figure 6b) and enhances the percentage of activated CD69+CD25+ T cells (Figure 6a), but decreases percentage of inflammatory IL-17+ and IFN-γ+ T cells (Figure 6c,d). Since DPPS enhances percentage of Tregs in the liver and decreases percentage of inflammatory T cells, it can be assumed that the observed protective role of DPPS in ConA-induced hepatitis can be also mediated by alteration of immune response in the liver. In accordance with enhanced percentage of Tregs in the livers of DPPS-treated and DPPS-pretreated ConA hepatitis mice, there is a higher percentage of IL-10-expressing F4/80 macrophages and CD11c dendritic cells in the livers of DPPS-treated mice (Figure 2) and higher expression of IL-10 in liver tissue at the protein level (Figure 5b), since it is known that IL-10 induces regulatory phenotype of T cells [52]. There are results that report opposite effects of different oganosulfur compounds on IL-10 production [14]. However, treatment with garlic extract significantly increases production of IL-10, while decreases TNF-α production in LPS-stimulated placental cells [53]. Thus, it can be concluded that protective effects of DPPS in ConA-induced hepatitis could be also mediated by altering Treg/Th17 balance in favor of Tregs.

In summary, our study demonstrates that DPPS can protect mice against acute inflammatory liver injury induced by ConA. The protective effect of DPPS is associated with attenuation of hepatocyte apoptosis, production of inflammatory mediators, and intrahepatic leukocyte recruitment, shifting the balance between pro- and anti-oxidative enzymes in favor of antioxidative, and by stimulation of Tregs in the liver. Consequently, our findings highlight the mixture of DPPS as a potential therapeutic agent that could have hepatoprotective effects in acute liver inflammatory diseases.

## Figures and Tables

**Table 1:** Primers used for Real-Time qRT-PCR.

Target	Sense and Antisense
ICAM-1	forward 5′-CAATTTCTCATGCCGCACAG-3′, reverse 5′-AGCTGGAAGATCGAAAGTCCG-3′
VCAM-1	forward 5′-TGAACCCAAACAGAGGCAGAGT-3′, reverse 5′-GGTATCCCATCACTTGAGCAGG-3′),
PECAM-1	forward 5′-CAAACAGAAACCCGTGGAGATG-3′, reverse 5′-ACCGTAATGGCTGTTGGCTTC-3′
P-selectine	forward 5′-TCATCCCGGTGAAGCAATGT3′, reverse, 5′-TGGAGAACGCAAGGACAGGTAT-3′
p47^phox^	forward 5′-ATGACCTCAATGGCTTCACC-3′, reverse 5′-CTATCTGGAGCCCCTTGACA-3′
p67^phox^	forward 5′-CTATCAGCTGGTTCCCACGA-3′, reverse 5′-GCAGTGGCCTACTTCCAGAG-3′
iNOS	forward 5′-CGAAACGCTTCACTTCCAA-3′, reverse 5′-TGAGCCTATATTGCTGTGGCT-3′
SOD1	5′-AATGTGTCCATTGAAGATCGTGTGA-3′, reverse 5′-GCTTCCAGCATTTCCAGTCTTTGTA-3′
SOD2	forward 5′-AGGGCCTGTCCCATGATGTC-3′, reverse 5′-AGAAACCCGTTTGCCTCTACTGAA-3′
SOD3	forward 5′-GGGTCTGTCCTGTACTTCACCAGAG-3′, reverse 5′-CTGACATGGTCCAGGTGACAGAG-3′
GAPDH	forward 5′-CATCACTGCCACCCAGAAGACTG-3′, reverse 5′-ATGCCAGTGAGCTTCCCGTTCAG-3′

## Data Availability

Data described in the manuscript will be made available upon reasonable request from the corresponding author.
